# *Mycoplasma gallinarum* infection associated with apparent *Mycoplasma meleagridis* seropositivity in breeder turkey flocks

**DOI:** 10.2478/jvetres-2026-0041

**Published:** 2026-08-06

**Authors:** Joanna Kowalczyk, Marcin Śmiałek, Olimpia Kursa

**Affiliations:** 1 Department of Poultry Diseases, Faculty of Veterinary Medicine, University of Warmia and Mazury, 10-687 Olsztyn, Poland; 2 SLW BIOLAB, ul. Grunwaldzka 42, 14-100 Ostróda, Poland; 3 Department of Bacteriology and Bacterial Animal Diseases, National Veterinary Research Institute, 24-100 Puławy, Poland

**Keywords:** *Mycoplasma* spp., breeder turkey, cross-reactivity, serological methods

## Abstract

**Introduction:**

Monitored EU-wide, mycoplasmosis is a significant poultry disease caused by bacteria of the genus *Mycoplasma*, among which *M. gallisepticum, M. synoviae, M. meleagridis* and *M. iowae* are of major importance. Infection can lead to clinical disease and economic losses. Serological assays can give false positive results because of antigenic cross-reactivity among *Mycoplasma* species. Molecular diagnostics provide greater specificity and reliability. This study investigated suspected cases of *Mycoplasma meleagridis* infection in turkeys and assessed whether the observed serological reactivity was by cross-reactive antibodies rather than a sign of *M. meleagridis* infection.

**Material and Methods:**

One case in a breeder turkey flock was investigated during the rearing period, and another case in a similar flock was explored in the laying period. Both flocks had serology results positive for *M. meleagridis*. The rearing flock was monitored with ELISA, PCR and sequencing for extended time after a single positive result, whereas the laying flock was testing using rapid plate agglutination (RPA) in addition because mortality was higher and egg production lower. In both cases, swab and blood samples were taken to verify the initial serological findings and assess the potential presence of *M. meleagridis*.

**Results:**

In the breeding flock, ELISA detected a single positive reaction against *M. meleagridis* in one of three houses. However, a PCR did not confirm the presence of *M. meleagridis* DNA, although *Mycoplasma* spp. DNA was detected at low/inconclusive levels. In the laying flock, the RPA test gave several seropositive reactions against *M. meleagridis*, whereas a PCR did not confirm its presence. Microbiological culture, 16S rRNA PCR and Sanger sequencing identified *M. gallinarum* in both flocks.

**Conclusion:**

The results highlight the limited specificity of serological assays and the necessity of molecular confirmation for accurate diagnosis.

## Introduction

Mycoplasmosis is one of the most important poultry diseases that poses a global epidemiological and economic challenge. It is caused by small bacteria lacking a cell wall, belonging to the phylum *Mycoplasmatota*, class *Mollicutes* and family *Mycoplasmataceae*. To date, more than 27 *Mycoplasma* species have been described in birds, and they can affect a wide variety of avian hosts, including chickens, turkeys, house finches, songbirds, game birds, peafowl, Japanese quail, bobwhite quail, pigeons, ducks and geese. However, both nationally and worldwide only four species – *M. gallisepticum* (MG), *M. synoviae* (MS), *M. meleagridis* (MM) and *M. iowae* (MI) – play a significant role in poultry pathology (16, 21). Infection occurs through direct contact between susceptible birds and infected carriers, *via* contaminated feed or water, or through vertical (transovarian) transmission from breeder hens to their offspring (18). *Mycoplasma* spp. exhibit tropism for specific organ systems, including the respiratory and urogenital tracts, conjunctiva, sinuses, joints, and occasionally the gastrointestinal tract (1, 2).

Alongside pathogenic *Mycoplasma* species, non-pathogenic species have also been identified, including *M. gallinarum* (MGn), which is considered a commensal not only in birds but also in other hosts, such as pigs, goats and sheep (11). Although relatively little is known about MGn, it has been demonstrated that infection is associated with the delayed onset of fatty liver haemorrhagic syndrome in laying hens (4).

Pathogenic *Mycoplasma* species are generally considered microorganisms of relatively low virulence in the context of single-agent infections, and the development of clinical disease in poultry typically requires the presence of additional predisposing factors. Among the four significant *Mycoplasma* spp., only MG is capable of independently inducing clinical disease in turkeys, which manifests as infraorbital sinusitis. In the case of transovarian infection with MM, it is established that hatchability declines and hatched poults exhibit respiratory signs and are of low quality (8).

*Mycoplasma* infections have caused substantial economic losses, particularly in the USA and Europe. Consequently, to limit the spread of this pathogen, several countries in the European Union have implemented monitoring and control programmes for infections in breeding poultry. According to the legislation currently in force in Poland, breeding flocks of turkeys and chickens are subject to mandatory monitoring for infection with MG (turkeys and chickens) and MM (turkeys), enabling continuous surveillance of the epizootic situation in Poland. Monitoring is based on serological and/or bacteriological testing of blood samples, semen or swabs collected from the trachea, cloaca or air sacs of turkeys from the first day of life until the end of their production. Detection of *M. gallisepticum* and/or *M. meleagridis* is attempted in a representative number of samples in order to enable continuous surveillance of infection during the rearing and laying periods, whereby flocks are sampled immediately before the onset of egg production and subsequently every three months throughout the production cycle. This scheme also monitors and interrupt vertical transmission (5).

Laboratory diagnosis of *Mycoplasma* infections using serological methods may sometimes be challenging and not entirely conclusive. As early as the 1970s, reports described the occurrence of false-positive results in the haemagglutination inhibition test, attributed to cross-reactivity between *Mycoplasma* species (24). Subsequent studies have demonstrated the presence of shared genomic sequences and antigenic epitopes between MG and MS, which may explain the cross-reactivity observed in routine serological assays. Moreover, the use of tests based on the detection of whole-cell antigens increases the frequency of both cross-reactive and nonspecific reactions (6, 7, 27). Interestingly, cross-reactivity has been observed not only among avian *Mycoplasma* species but also among species infecting other animals, including pigs (19). Because the interpretation of serological results is limited, diagnostic methods based on molecular biology and offering high sensitivity and specificity are considered ideal tools. These methods enable the detection of *Mycoplasma* isolates, genotyping and the differentiation between field and vaccine strains (3, 9, 10). This study aimed to investigate whether apparent MM seropositivity in breeder turkey flocks could be associated with infection by other *Mycoplasma* species.

## Material and Methods

### Case 1 – breeder turkey flock during the rearing period

The flock was raised in three production houses (H1, H2 and H3). Suspected samples were collected at 22 weeks of age (May 2025) in accordance with the schedule of the farm’s internal flock health monitoring programme. Blood samples (n = 23 per house) were collected from randomly selected birds in all the houses for serological testing. One of the diagnostic targets was the detection of specific antibodies against *M. meleagridis*. The results were negative for H1 and H2, but one was positive for H3. In response to this finding, extended monitoring of *M. meleagridis* was implemented. At 24 weeks of age, blood samples (n = 23 per house) were collected again for serological examination. In addition, 60 tracheal swabs were collected from turkeys in house H3, and 30 tracheal swabs were collected from each of houses H1 and H2. Serum was obtained from blood samples and tested using ELISA. All swabs were collected in duplicate; one set was used for PCR testing (swabs were pooled, 10 swabs per one pooled sample), while the second set was submitted for sequencing at the National Veterinary Research Institute in Puławy, Poland. At 24 weeks of age, the turkeys received targeted antimicrobial therapy against *Mycoplasma* infections in the form of tylosin administered in drinking water. At 26 weeks of age, the turkeys had blood sampled (n = 23 per house) again for serological testing; 30 tracheal swabs from birds in each production house (pooled, 10 per composite sample) were also taken (Fig. 1 Case 1).

**Fig. 1.**
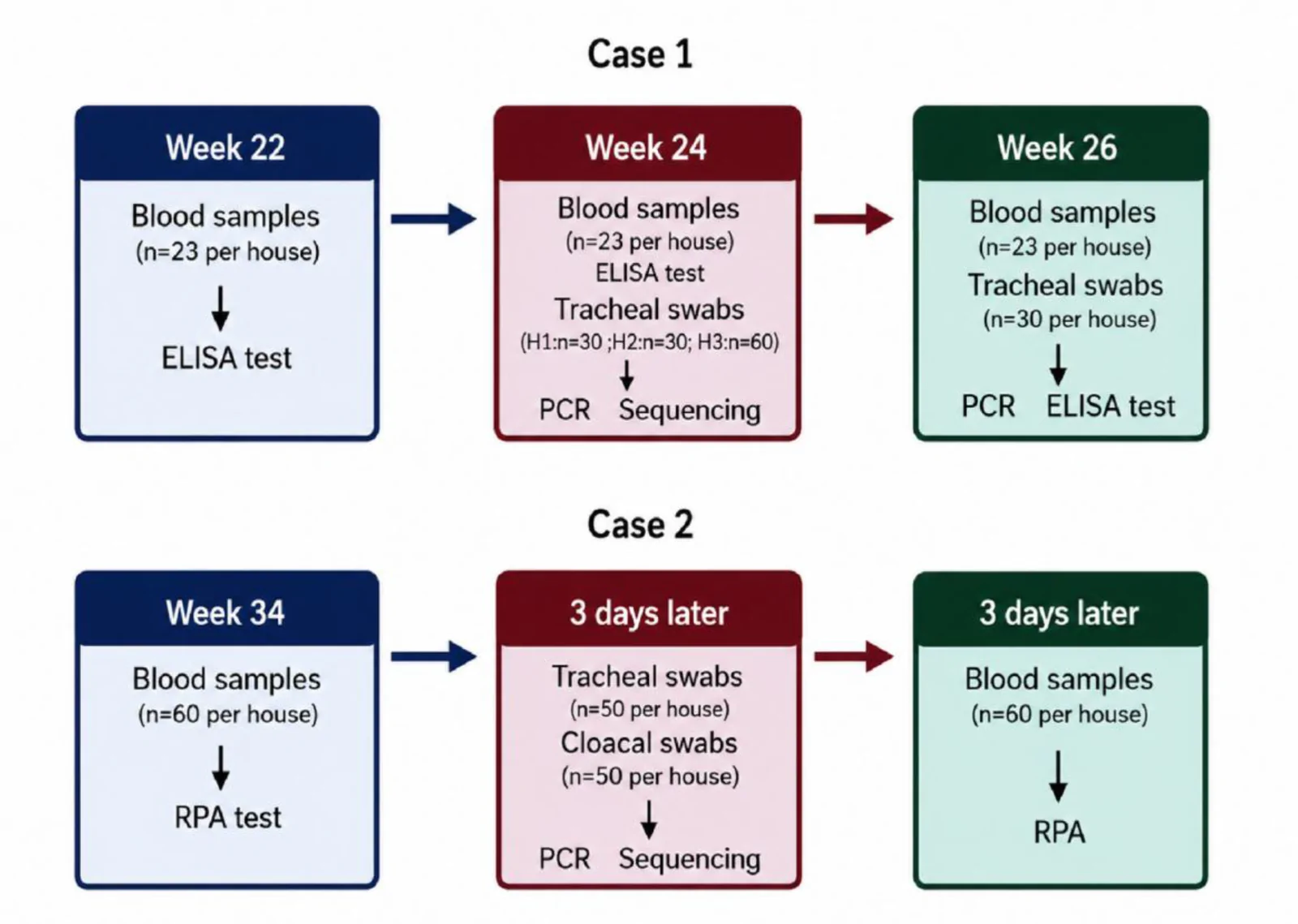
Sampling time points and types of samples collected from turkeys for *Mycoplasma* infection testing

### Case 2 – breeder turkey flock during the laying period

The flock was raised in four production houses (H1–H4). The first suspected samples were collected at 34 weeks of age (June 2025). Blood samples were collected from randomly selected birds in all houses (n = 60 per house) and tested using rapid plate agglutination (RPA) to detect antibodies against MM. An increase in mortality and a decrease in egg production was observed in the flock, prompting additional laboratory investigations. These revealed infection with *Ornithobacterium rhinotracheale* in the birds; appropriate treatment was implemented, and no association with *Mycoplasma* was found. On the day the RPA results were obtained, tracheal swabs (n = 50 per house) and cloacal swabs (n = 50 per house) were collected in duplicate from birds in each production house. Ten swabs were pooled into one sample and submitted to PCR testing targeting *Mycoplasma* spp. and MM. The second set of swabs was used for genetic sequencing analysis. Three days after obtaining the positive RPA results, an additional 60 blood samples were collected from birds in each production house for repeat testing using RPA to detect antibodies against MM (Fig. 1, Case 2).

### ELISA test

*Mycoplasma meleagridis* serological evaluations in bird serum samples were performed using commercial ELISA kits (IDEXX, Westbrook, ME, USA). The successive steps of the ELISA tests were performed according to the manufacturer’s recommendations. The ELISAs were carried out using an Eppendorf epMotion 5075 LH automated pipetting station (Eppendorf, Hamburg, Germany), ELx405 automatic plate washer and ELx800 plate reader (BioTek, Winooski, VT, USA). Internal positive controls (GD Animal Health, Deventer, the Netherlands) were incorporated to validate the test results. For individual samples, the sample-to-positive values were calculated based on the optical densities of the sample and positive and negative controls. The sample-to-positive values were used to calculate the antibody titres for individual samples in the software provided by IDEXX for the interpretation of their ELISA tests. The kit manufacturers’ standard cut-off values were applied in order to interpret the samples as negative or positive. The individual titres of MM antibodies for each sample were used to express the mean geometric titre (Gmean) for the evaluated sample batches.

### RPA test

This test was performed to detect antibodies against MM. Blood samples were collected from randomly selected birds, and the serum was aseptically separated within 24 h of collection and stored at –20°C until analysis. Prior to testing, serum samples and antigens were brought to room temperature (20–25°C). The RPA test was performed using commercially available coloured antigens (MM RPA-Test; Ceva Biovac, Beaucouzé, France), according to the manufacturer’s instructions. Briefly, 25 μL of serum (undiluted and diluted 1:4) was placed on a clean glass plate and mixed with an equal volume of antigen using a micropipette tip. The plate was gently rotated to ensure proper mixing, and the reaction was observed for up to 3 min. The presence of distinct blue-violet agglutinates (clumps) which appeared within 3 min was interpreted as a positive result, and the absence of visible agglutination after this time was considered negative.

### PCR

After collection, 10 pooled tracheal swabs were suspended in phosphate-buffered saline (PBS) for PCR. Total bacterial genetic material from the prepared samples was isolated using a commercial MagMAX CORE Nucleic Acid Purification Kit (Life Technologies/Thermo Fisher Scientific, Carlsbad, CA, USA) and a KingFisher Duo Prime Purification System (Life Technologies/Thermo Fisher Scientific). The resulting nucleic acid extracts were used to detect the presence of *Mycoplasma* DNA in a commercial real-time RT-PCR kit (EURx, Gdańsk, Poland). A Bio-X PCR kit was used to detect and differentiate *M. meleagridis* and *M. iowae* (Bio-X Diagnostics, Rochefort, France). Real-time RT-PCR reactions were performed according to the manufacturer’s protocol using a 7500 CFX96 Real Time System thermocycler (Bio-Rad, Hercules, CA, USA). Fluorescence results were obtained using the dedicated CFX Manager Dx software (Bio-Rad).

### *Mycoplasma* isolation and sequencing

Ten pooled tracheal swabs were initially incubated in Avian Mycoplasma Liquid Medium (Mycoplasma Experience, Bletchingley, UK) at 37°C for 3 h. Each sample was inoculated onto pleuropneumonia-like organism (PPLO) agar plates and incubated at 37°C under a 5% CO_2_ atmosphere. The plates were examined every 2–3 d for the presence of *Mycoplasma* colonies in the culture. Upon detection of typical colonies, single colonies were subcultured into fresh liquid medium and incubated at 37°C. Extraction of DNA was carried out from 200 μL of broth culture using the QIAamp DNA Mini Kit (Qiagen, Hilden, Germany) according to the manufacturer’s instructions, and the DNA was stored at -20°C until further analysis. *Mycoplasma* spp. was identified using a modified PCR assay targeting the 16S ribosomal RNA (rRNA) gene. Amplification was performed using primers previously described by Lierz (12) with minor modifications. The reaction conditions, including reagent concentrations and thermal cycling parameters, were those of the protocol reported by Sawicka *et al*. (22). The expected PCR products were approximately 900 bp in length. Representative PCR amplicons obtained from isolates originating from poultry house H3 (Case 1) and poultry houses H1 and H3 (Case 2), which were positive in both serological testing and PCR, were subjected to Sanger sequencing (Genomed, Warsaw, Poland) to confirm the species identity of the isolates. Sequence analysis was performed using the BLASTn (basic local alignment search tool, nucleotides) algorithm against the NCBI GenBank nucleotide database. Species identification was based on the highest sequence similarity to the reference *Mycoplasma* sequences available in GenBank.

### Statistical analysis

Owing to the descriptive case report nature of this study, no statistical analyses were performed.

## Results

### Case 1 ELISA test

The ELISA results for field case 1 are summarised in Table 1. The results were negative in H1 and H2, whereas in H3, one positive result for *M. meleagridis* was detected at 22, 24 and 26 weeks of life. For house H3, the Gmean values at weeks 22, 24 and 26 were 275, 251 and 285, respectively.

**Table 1.** Results of ELISA tests for *Mycoplasma meleagridis* in a breeding turkey flock in the rearing period

Poultry house	Week 22	Week 24	Week 26
H1	negative (23/23; 100%)	negative (23/23; 100%)	negative (23/23; 100%)
H2	negative (23/23; 100%)	negative (23/23; 100%)	negative (23/23; 100%)
H3	positive (1/23; 4%)	positive (1/23; 4%)	positive (2/23; 9%)

### Case 1 PCR

The molecular testing results are presented in Table 2. No *Mycoplasma meleagridis* genetic material was detected in any of the samples collected from birds in houses H1–H3, either at 24 weeks of age or after tylosin treatment at 26 weeks of age. Assays targeting *Mycoplasma* spp. were negative in houses H1 and H2 at both sampling time points, whereas in H3, a positive/inconclusive result was obtained at week 24 and an inconclusive result at week 26.

**Table 2.** Results of real-time PCR to amplify *Mycoplasma* DNA in samples from a breeding turkey flock in the rearing period

Poultry house	Week 24 *Mycoplasma meleagridis*	Week 24 *Mycoplasma* spp.	Week 26 *Mycoplasma meleagridis*	Week 26 *Mycoplasma* spp.
H1	negative (3/3; 100%)	negative (3/3; 100%)	negative (3/3; 100%)	negative (3/3; 100%)
H2	negative (3/3; 100%)	negative (3/3; 100%)	negative (3/3; 100%)	negative (3/3; 100%)
H3	negative (6/6; 100%)	positive/inconclusive (6/6; 100%) (Ct 35.1; 35.3; 37; 37.3; 38; 38.7)	negative (3/3; 100%)	inconclusive (1/6; 17%) (Ct 39)

1 Ct – threshold cycle

### Case 2 RPA test

The RPA test revealed two positive samples (both without dilution and after a fourfold dilution) and one sample that was positive without dilution but negative after dilution in house H1. In house H3, one sample was positive both without dilution and after fourfold dilution, and one sample was positive without dilution but negative after dilution. No positive results for MM were detected in houses H2 or H4.

### Case 2 PCR

The results of molecular testing are summarised in Table 3. No *M. meleagridis* genetic material was detected in any of the samples collected from birds in houses H1–H4. Assays targeting *Mycoplasma* spp. were negative in tracheal swabs from H3 and H4, whereas positive/inconclusive results were obtained from H1 and H2.

**Table 3.** Results of real-time PCR to amplify *Mycoplasma* DNA in samples from a breeding turkey flock in the laying period

Poultry house	*Mycoplasma* spp. in cloacal swabs	*Mycoplasma* spp. in tracheal swabs	*Mycoplasma meleagridis* in cloacal swabs	*Mycoplasma meleagridis* in tracheal swabs
H1	positive (3/5; 60%) (Ct 34.6, 36.1, 36.3)	positive/inconclusive (3/5; 60%) (Ct 34.2, 37.7, 38.2)	negative (5/5; 100%)	negative (5/5; 100%)
H2	positive (4/5; 80%) (Ct 32.1, 33.9, 34.1, 35.5)	positive/inconclusive (4/5; 80%) (Ct 36.7, 36.8, 37.1, 38.0)	negative (5/5; 100%)	negative (5/5; 100%)
H3	positive (4/5; 80%) (Ct 29.6, 33.9, 36,5, 36.8)	negative (5/5; 100%)	negative (5/5; 100%)	negative (5/5; 100%)
H4	positive/inconclusive (3/5; 60%) (Ct 36.0, 37.1, 37.9)	negative (5/5; 100%)	negative (5/5; 100%)	negative (5/5; 100%)

1 Ct – threshold cycle

### Isolation and sequencing of *Mycoplasma* spp

In both herds, colonies with morphology typical of *Mycoplasma* spp. were observed on PPLO agar during incubation. The colonies appeared within the incubation period and were successfully subcultured. Amplification targeting the 16S rRNA gene confirmed the presence of *Mycoplasma* spp. DNA from the obtained cultures. In positive samples, an amplicon of the expected approximate 900-bp size was detected, confirming the isolates as *Mycoplasma gallinarum*. Representative PCR products obtained from three isolates originating from H3 (Case 1) and H1 and H3 (Case 2), which were the houses in which positive serological and PCR results were obtained, were subjected to Sanger sequencing. Analysis of an 866-bp sequence fragment with BLAST showed 99.08–99.19% sequence identity with reference MGn 16S rRNA gene sequences deposited in GenBank with 100% query coverage (E-value = 0.0). These findings confirmed that the isolates belonged to *Mycoplasma gallinarum*. The sequencing results are presented in Table 4.

**Table 4.** Results of Sanger sequencing and BLAST analysis of *Mycoplasma gallinarum* isolates from breeding turkey flocks in the rearing and laying periods

Flock period	Poultry house	Species identified	Sequence identity (%)	Query coverage (%)	Closest GenBank accession No.
Rearing	H3	*Mycoplasma gallinarum*	99.19	100	MH538998.1
Laying	H1	*Mycoplasma gallinarum*	99.08	100	MH538998.1
Laying	H3	*Mycoplasma gallinarum*	99.08	100	JN935884.1

## Discussion

In the laboratory diagnosis of bacterial infections, particularly those included in national monitoring programmes, the selection of an appropriate diagnostic method is critical. Based on the analysis of field cases, it has been demonstrated that positive serological results for MM may result from cross-reactivity with MGn rather than from a true MM infection.

The cross-reactivity observed in both the RPA and ELISA assays confirms the previously described limitations of serological diagnostics in *Mycoplasma* infections. As early as the 1970s, Vardaman *et al*. (24) reported false-positive results in the haemagglutination inhibition test attributable to cross-reactions among *Mycoplasma* species. Published studies indicate that this phenomenon is not restricted to avian mycoplasmas but occurs across *Mycoplasma* species irrespective of the host and has been extensively described in swine and humans (12, 19, 23). In the 1980s, Lind *et al*. (13) demonstrated cross-reactivity between *M. genitalium* and *M. pneumoniae* in samples collected from men with nongonococcal urethritis. Despite the relatively low genetic similarity between these two species (approximately 1.8% homology in base sequences), shared epitopes and substantial similarity in antigenic responses across different serological assays have been identified. These findings indicate that cross-reactivity represents a significant diagnostic limitation that may lead to misinterpretation of laboratory test results. Consequently, the use of molecular methods as confirmatory tools is strongly recommended, particularly in cases of epidemiological significance.

As in the cases described previously, the present study also demonstrated potential serological cross-reactivity among *Mycoplasma* species. Although serological testing yielded positive results for MM, subsequent molecular analyses and sequencing excluded infection with this agent and confirmed the presence of an alternative species, MGn.

A comparative analysis of serological methods used in the diagnosis of *Mycoplasma* infections in poultry, particularly for the detection of MG and MS antigens, clearly demonstrates differences in the sensitivity and specificity of individual assays, as well as the potential occurrence of non-specific and cross-reactions (6, 7, 17, 26). Yadav *et al*. (26) reported a higher number of seropositive samples for MG and MS when using an indirect ELISA than when using a serum plate agglutination test and dot ELISA, indicating the higher sensitivity of this method in the serodiagnosis of birds exhibiting respiratory symptoms (26). The variability in the sensitivity and specificity of laboratory diagnostic methods, along with the results of our field studies, further highlights the limitations of relying on a single diagnostic assay and supports the use of complementary testing strategies for the accurate identification of *Mycoplasma* spp.

The available literature indicates that cross-reactivity between MG and MS primarily results from the presence of conserved epitopes of surface lipoproteins and functional domains of adhesins (*e.g*. GapA and VlhA) (23, 27). Therefore, in conventional ELISA tests based on whole-cell antigens or membrane proteins, reactions with antibodies directed against heterologous *Mycoplasma* species are frequently observed, further complicating the interpretation of seropositive samples. These findings are consistent with our observations of moderate cross-reactivity of MGn in RPA and ELISA assays in the context of suspected MM exposure.

*Mycoplasma gallinarum* is generally regarded as a commensal species or microorganism of low pathogenicity; however, recent molecular studies have indicated that its biological and diagnostic significance may be greater than previously assumed (21, 25). Owing to its antigenic similarity to other *Mycoplasma* species occurring in poultry, its presence in birds may lead to false-positive results in serological tests, including those targeting MG and MM, which are included in national monitoring programmes. Consequently, this may result in an incorrect assessment of flock health status and the implementation of unnecessary administrative measures. To avoid nonspecific and cross-reactions in the serodiagnosis of *Mycoplasma* infections, methods with higher sensitivity and specificity should be employed, including more specific serodiagnostic assays. Examples include ELISA based on recombinant proteins, such as the MS087 protein (28) or recombinant P50 protein-based assays (14), for the diagnosis of *M. synoviae*.

The present study has several limitations. First, the number of field cases available for investigation was limited, which restricted the generalisability of the findings. Furthermore, comprehensive laboratory analyses, including detailed antigenic characterisation of individual isolates, were not performed. Consequently, the potential role of antigenic similarities in contributing to serological cross-reactivity could not be directly assessed. Moreover, the involvement of other factors capable of nonspecific serological reactions cannot be excluded. Therefore, additional studies based on larger case series and more extensive phenotypic and molecular characterisation of isolates are warranted to confirm and further elucidate the mechanisms underlying these findings.

Seropositive results must always be confirmed using molecular methods, as these techniques are highly specific, and the likelihood of false results is significantly minimised. Furthermore, PCR and sequencing enable the differentiation and precise identification of isolated strains (6, 7, 15, 17, 20, 21).

## Conclusion

The analysis of field cases and the obtained molecular results confirmed the presence of MGn, thereby excluding infection of the birds with MM despite seropositive findings. This strongly suggests that MGn infection may contribute to serological reactivity interpreted as MM exposure, and highlights the necessity of molecular confirmation to ensure an accurate diagnosis.
